# Multiple lung abscesses due to acinetobacter infection: a case report

**DOI:** 10.1186/1757-1626-2-9347

**Published:** 2009-12-18

**Authors:** Ioannis Kokkonouzis, Ioannis Christou, Ioannis Athanasopoulos, Nikolaos Saridis, Vasilios Skoufaras

**Affiliations:** 1 Department of Respiratory medicine, Hellenic Air Force General Hospital, Athens, Greece; 2 3rd Internal Medicine Department, Hellenic Air Force General Hospital, Athens, Greece

## Abstract

Acinetobacter species are well-known causes of nosocomial infections. Recent increasing evidence emphasize on the role of these pathogens in community-acquired infections.

We report a case of a 16-yr-old female with fever, sore throat, productive cough, malaise and the presence of lung consolidation with multiple abscesses on radiographic examination. The patient had no significant medical history. After a detailed diagnostic work-up the diagnosis of community acquired Acinetobacter pneumonia with multiple lung abscesses was made. The Acinetobacter stain was susceptible to a variety of antimicrobial agents and the patient's condition improved rapidly. A new computed tomography chest scan, three months later, confirmed full recovery.

The presence of lung abscesses due to Acinetobacter infection is an extremely uncommon manifestation of the disease. This case underlines the emergent role which these, often multi-drug resistant, bacteria may play in the future, perhaps in community infections as well.

## Case presentation

A 16-yr-old female presented to the emergency department with a two-day history of fever up to 39°C, sore throat, productive cough and malaise. Initially a diagnosis of acute tonsillitis was made and the patient was treated for a seven-day period with penicillin. Unfortunately the patient remained febrile up to 38°C, with persistent productive cough and malaise. Ten days after her first visit she was admitted to the pulmonary department for further evaluation. The patient until then had no significant medical history.

**Figure 1:**
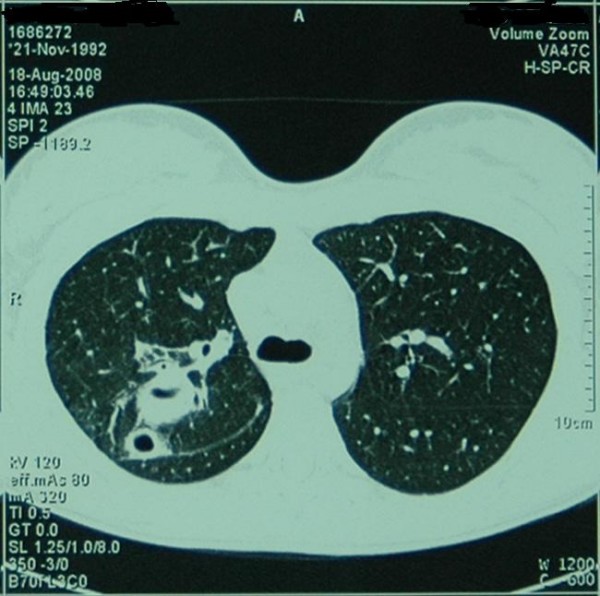
**The computed tomography scan of this 16-y- old female revealed the presence of multiple abscesses at the right upper lobe**.

On admission she had low-grade fever (37,4°C). The rest of the clinical findings were unremarkable. Blood tests demonstrated normal white cell count (9500/μl), anemia (hematocrit 32,6% and hemoglobin 10,7 gr/dl), mild thrombocytosis (512000/μl), increased erythrocyte sedimentation rate (ESR = 109 mm/hr) and C-reactive protein (CRP = 49,6 mg/dl). Serum biochemistry tests were within normal limits. Arterial blood gases were normal as well. The chest radiograph disclosed a right upper lobe consolidation with the presence of at least three abscesses within the lobe. A spiral computed tomography of the chest demonstrated the consolidation within the right upper lobe with multiple lung abscesses (Figure 1). No other evidence of parechymal damage, lymph nodes enlargement or pleural fluid was visible. Heart and great vessels visualized without any pathology.

**Figure 2:**
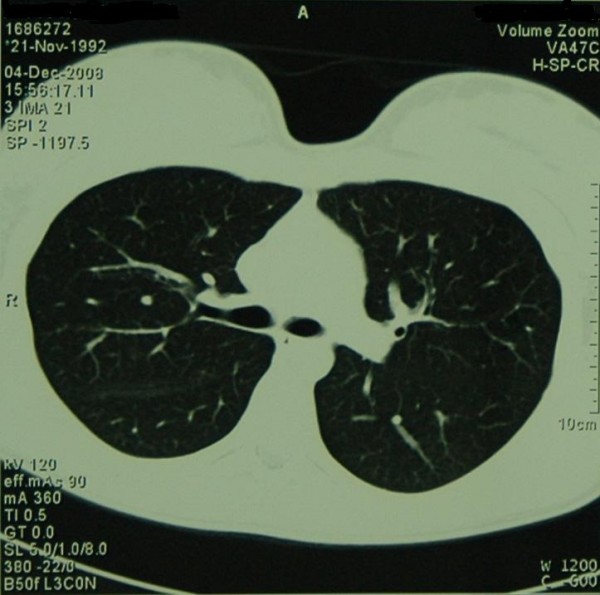
**After a three-month period of treatment a new computed tomography scan demonstrated a full recovery**.

Cardiological examination including heart ultrasonographic analysis and dental examination did not demonstrate any pathological evidence. Skin tests for M. tuberculosis, atypical mycobacteria and Quanti-FERON TB Gold test were negative. Serum component analysis, rheumatoid factor, antinuclear antibodies, antineutrophilic cytoplasmic antibodies, immunonoglobin levels, serologic tests for hepatitis A, B, C, D, human immunodeficiency virus and common viruses did not reveal any abnormality. Finally sputum cultures in three different samples revealed the presence of Acinetobacter baumannii and the diagnosis of community-acquired pneumonia with multiple abscesses due to Acinetobacter baumannii infection were made based on this evidence. The stains were susceptible to multiple antibiotics and treatment with ciprofloxacin and amoxicillin with clavoulanic acid was initiated for a three month period of treatment. A significant clinical improvement in the patient's condition was noted only a few days later which was reflected in her laboratory investigation as well. Three months later a new computed tomography scan of the chest was obtained revealing an almost completely healed right upper lobe (Figure 2). The patient remains asymptomatic until now.

## Discussion

Acinetobacter spp are aerobic gram-negative coccobacillus with preference for warm, ambient environments, which have received a great interest during the last two decades due to its ability to accumulate, with multiple mechanisms, multi-drug resistance stains. As a result these bacteria have become, a sometimes lethal, etiologic factor mainly in intensive care unit (ICU) infections, ventilator associated pneumonia (VAP), and in community-acquired infections and represent an emergent public health problem [1,2].

Typically this pathogen has been described as a cause of infection in tropical or subtropical climates, more often during summer, in ICU patients or in long term residents at health care facilities. Ventilator-depended patients are at greater risk but other factors such as recent surgery, central vascular lines, tracheotomy, enteral feeding and treatment with new generation antibiotics, like third-generation cephalosporins, fluoroquinolones or carbapenems, may contribute as well. The majority of affected patients have underlying comorbidities mainly chronic obstructive pulmonary disease, renal disease, diabetes mellitus, alcoholism and heavy smoking [1,3,4].

Nosocomial outbreaks have also been described due to health care professionals with colonized hands and poor personal hygiene. These people could act as opportunist carriers of an epidemic stain. Contaminated ventilators or respiratory care equipment as well as intrahospital transmission may also contribute to the beginning of an outbreak [1,4].

Recently Turkey's earthquakes in Marmara area, as well as others physical disasters like the South Asia tsunami, demonstrated that vulnerable populations are also at risk to Acinetobacter infections due to contamination and cross-infection inside hospital settings [1]. Moreover as a result of the ongoing war conflicts in Afghanistan and Iraq some cases of multi-drug resistant Acinetobacter outbreaks have also been reported bringing reminiscent of similar reports during the Korean and Vietnam wars. It seems that in cases like these multiple factors can contribute including contamination of wounds in the battlefield, local food, environmental spread and cross-infection in the local military or abroad hospitals [5].

Community-acquired infections due to these pathogens are still rarely reported, mainly in warm and humid climates, and pneumonia. Fulminant course with an acute onset of dyspnea, cough, and fever, is the most common manifestation. Most patients suffer from various comorbidities. Leung et al in a three- year retroprospective study involving 19 patients with community-acquired pneumonia (CAP) due to Acinetobacter baumannii and 74 patients with health care associated pneumonia (HAP) concluded that CAP appears to be a unique clinical entity with a high incidence of bacteremia, ARDS, disseminated intravascular coagulation (DIC), and death, when compared to HAP [6]. In another study involving thirteen patients Chen et al concluded that Acinetobacter baumannii can be considered as an etiologic factor in community-acquired lobar pneumonia in patients with fulminant course in the warmer and humid periods of the year and also in younger alcoholic patients. The authors also suggested that a good sputum smear originated from the lower respiratory tract if it contains >25 leukocytes per high power (100×) field on microscopic examination early on to establish the diagnosis and that a combination of a third-generation cephalosporin and an aminoglycoside is adequate as empirical treatment [7]. Falagas et al in a recent review highlighted six case series with community-acquired Acinetobacter infections involving 80 patients. Of these 51 had pneumonia and 56% (45 patients) died. Moreover in the same review 26 case reports involving 43 patients had been identified. Pneumonia was present in 38 of them and from the others two had meningitis, one had native valve endocarditis, one had soft skin infection and one had an ocular infection. Chronic obstructive pulmonary disease, renal disease, diabetes mellitus, excess consumption of alcohol and heavy smoking were the most common risk factors. According to the same review on 12 retrospective or prospective studies the range of isolation of Acinetobacter from patients suffering from community-acquired pneumonia was 1, 3% to 25, 9% [8].

A variety of antibiotics can be used if the infection has been caused by a susceptible stain including broad-spectrum cephalosporins, β-lactam-β-lactamase inhibitor combinations, carbapenes, and fluoroquinolones alone or in combination with aminoglycosides. Multi-drug resistant infections, including carbapenes, aminoglycosides and fluoroquinolones resistance, are challenging, however the combination of intravenous or inhaled colistin with tigercycline, imipenem, rifampicin or azithromycin seems reasonably enough to achieve satisfactory efficiency, at least in vitro, due to synergic or addictive effects which these combinations offer. On the other hand it's unclear if these antibiotic combinations provide adequate therapeutically results in vivo [1,2,9,10].

To our knowledge only three other cases of lung abscesses and three more of pneumatoceles due to Acinetobacter infection that have been described until now but none has been presented with multiple abscesses on admission. Myrianthefs et al reported a case of a 77-year old woman with multi-drug resistant Acinetobacter baumannii lung abscesses who underwent splenectomy after multiple traumas and remained for a long period in an ICU. Fortunately this patient recovered [11]. Hunt et al reported three cases of ICU pneumonia with the formation of pneumatoceles due to Acinetobacter infection. Unfortunately none of them survived [12]. Yen et al in a 23 cases retrospective review of pediatric lung abscesses reported only one case of secondary abscess due to Acinetobacter baumannii infection. Although only two deaths occurred in this group of patients it's unclear if one of them was the Acinetobacter baumannii infected patient [13]. Finally Yang et al reported a case of a 35-year-old male with right lobe necrotizing community-acquired pneumonia and lung abscess formation one week after his admission, who achieved a slow but fully recovery [14].

In conclusion we presented a case of right lobe community acquired pneumonia with the formation of multiple lung abscesses due to a susceptible Acinetobacter baumannii stain. This is a rare manifestation of a community acquired disease.

## Abbreviations

ESR: erythrocyte sedimentation rate; CRP: C-reactive protein; ICU: intensive care unit; VAP: ventilator associated pneumonia; CAP: community acquired pneumonia; HAP: health care associated pneumonia; ARDS: adult respiratory distress syndrome; DIC: disseminated intravascular coagulation.

## Consent

Written informed consent was obtained from the patient's legal guardian for publication of this case report and accompanying images. A copy of the written consent is available for review by the Editor-in-Chief of this journal.

## Competing interests

The authors declare that they have no competing interests.

## Authors' contributions

All authors equally contributed to this patient's treatment. Moreover IK and IA wrote the manuscript and VS had the overall responsibility of the respiratory department and the final approval of the manuscript.
